# Correction: The impact of internet use in the digital era on public political trust in China—An empirical study based on CGSS 2021 data

**DOI:** 10.1371/journal.pone.0343673

**Published:** 2026-03-02

**Authors:** The PLOS One Editors

## Notice of Republication

This article [1] was republished on February 9, 2026, to address an issue identified post-publication. Please download this article again to view the correct version.
