# Supplementary material for: The impact of internet use in the digital era on public political trust in China—An empirical study based on CGSS 2021 data
Source: PLoS One. 2025 Dec 31;20(12):e0339999. doi: 10.1371/journal.pone.0339999 (PMC12755784; doi:10.1371/journal.pone.0339999)
Supplement: S1 File — (PDF) [file pone.0339999.s001.pdf]

Annex to the questionnaire

The Chinese General Social Survey 2021 data used in this paper comes from the publicly available Chinese National Survey Data Archive at http://www.cnsda.org/index.php?r=projects/view&id=65635422.

This website provides the original questionnaire and two data formats.

Nine questions from the questionnaire and related data were selected for the study, some of which belong to the category of demographic factors, and the specific values assigned to all variables are presented in Table 1 of the article. The original Chinese questionnaire can be accessed by logging onto the website. The questions used in this article are provided below for reference:

Among the questionnaire questions used in this paper are as follows:

A2.Gender [Interviewer's Record]

Male 1

Female 2

A3. What is your date of birth?

Record: [____|____|____|____]year, [____|____]month, [____|____]day

A5.What is your religion?

No religion......1

Religion

Buddhism 11

Taoism 12

Folk beliefs (worshipping Mazu, Guan Gong, etc.) 13

Islam/Muslimism 14

Catholicism 15

Christianity 16

Orthodox Christianity 17

Other Christianity 18

Judaism 19

Hinduism 20

Other (please specify: _______________) 21

A7a.Your current highest level of education is:

No education at all ......1 → skip to question A8a

Private school, literacy classes ...... 2 → Skip question A8a

Primary school 3

Lower secondary school 4

Vocational high school 5

General high school 6

Secondary school 7

Technical school 8

University speciality (adult higher education) 9

University speciality (regular higher education) 10

University undergraduate (adult higher education) 11

University undergraduate (regular higher education) 12

Postgraduate and above 13

Others (please specify: ________________) 14

A10. current political status is:

Member of the Masses......1

Communist youth league member 2

Democratic Party 3

Member of the Communist Party of China, joined the party in [____|____|____|____|____] year 4

Don't know 98

Refused to answer 99

A28_5. In the past year, what was your use of the Internet (including mobile phone access)?

Never......1

Seldom 2

Sometimes 3

Frequently 4

Very frequently 5

Don't know 98

Refuse to answer 99

A30_12. In the past year, have you often engaged in online activities

in your free time?

Everyday......1

Several times a week 2

Several times a month 3

Several times a year or less 4

Never 5

Don't know 98

Refuse to answer 99

A43e.On balance, it seems that in this current society, your own socio-economic status belongs to the:

Upper layer 1

Middle and upper layers 2

Middle layer...... 3

Middle and lower layers 4

Lower layer 5

Don't know 98

Refused to answer 99

P5_4.How much do you trust the National People's Congress (NPC)? 0 stands for "Don't trust at all", 10 stands for "Trust at all", please choose an appropriate number from 0-10.

0→10,

98...... cannot choose
