# Supplementary material for: The impact of internet use in the digital era on public political trust in China—An empirical study based on CGSS 2021 data
Source: PLoS One. 2025 Dec 31;20(12):e0339999. doi: 10.1371/journal.pone.0339999 (PMC12755784; doi:10.1371/journal.pone.0339999)
Supplement: Attachment — Submitted filename: [Response to Reviewers].docx [file pone.0339999.s003.docx]

Rebuttal Letter for Manuscript ID: PONE-D-25-01950

Part 1: Cover Letter to the Editor

Revised Manuscript Submission (Manuscript ID: PONE-D-25-01950) - Response to Reviewers' Comments

Dear editor,

Thank you for giving us the opportunity to revise your manuscript entitled “The Impact of Internet Use in the Digital Era on Public Political Trust in China—An Empirical Study Based on CGSS 2021 Data” (Manuscript ID: PONE-D-25-01950]. We sincerely appreciate the time and effort invested by you and the reviewers in evaluating our work. We found the reviewers' comments to be constructive, insightful, and invaluable for improving the quality and impact of our paper.

We have carefully considered all points raised by the reviewers and have made extensive revisions to the manuscript accordingly. A detailed, point-by-point response to each specific comment from each reviewer is provided in the attached document. The main categories of revisions we have undertaken include:

We uploaded the dataset used in our paper in accordance with your journal's requirements to verify the reproducibility of the results. We also modified the format of the abstract and the format of the first-level, second-level, and third-level titles.

Comprehensive Reference Updates: References have been checked, updated where necessary, and formatted according to journal guidelines. Our commentary on cited works is now more substantial and critical.

All changes made in the revised manuscript are clearly highlighted in yellow text (or tracked using 'Track Changes' in Microsoft Word). We firmly believe that these revisions have substantially elevated the manuscript's quality in terms of language, clarity, scientific depth, logical structure, figure presentation, and reference accuracy, effectively addressing the core concerns raised by the reviewers.

We respectfully request that you reconsider our revised manuscript for publication in PLOS ONE.

We are fully committed to further refining this work and are more than willing to undertake any additional revisions deemed necessary to meet the high standards of this esteemed journal.

Thank you again for your time and consideration. We look forward to your further guidance.

Sincerely,

Lu Fang

On behalf of all co-authors

University of Electronic Science and Technology of China

Part 2: Detailed Point-by-Point Response to Reviewers' Comments

Manuscript ID: PONE-D-25-01950

Title: The Impact of Internet Use in the Digital Era on Public Political Trust in China—An Empirical Study Based on CGSS 2021 Data

Response to Reviewer #1 Comments

We thank Reviewer #1 for their careful reading and constructive suggestions, which have greatly helped us improve the manuscript. Below are our point-by-point responses. All modifications are made using revision mode.

**Comment 1 (Reviewer #1):** “Not all parts of the theoretical section, hypotheses, data and conclusions align. The relationship between internet usage and political trust is discussed in the theoretical section, connected to hypotheses 2 and 4, and discussed in the conclusion section. However, the part on why respondents have not answered the survey question on political trust is not grounded in theory. This makes that hypothesis 1 and 3 seem to come out of nowhere and leave the reader to wonder why not answering the survey question about political trust would be linked to internet usage.”

**Response:** We sincerely agree. We have added relevant discussion in lines 197 to 207 to make the proposal of hypothesis 1 more reasonable. Here, we introduce the concept of political participation, identify the factors influencing political participation, and explain that the CGSS uses in-home interviews format, which can hinder responses to these questions due to low levels of trust between respondents and interviewers. We also cite relevant literature demonstrating that the development of the internet has heightened awareness of personal privacy and that social media users are more concerned with protecting their online information than non-users. Furthermore, in lines 240-246, we cite relevant research indicating that frequent internet use negatively impacts social trust, which is correlated with political trust. Furthermore, due to the CGSS survey format, internet users are reluctant to participate in political questions. Furthermore, based on the argument that algorithmic technology and internet use exacerbate the decline of traditional political participation, we propose Hypothesis 3.

**Comment 2 (Reviewer #1):** “These results show that as the public uses the Internet, more unofficial information and unverified information are obtained, which leads to lower level of political trust”. The authors do not measure the content of the Internet usage, so based on the data no conclusions can be drawn about the information that respondents receive online.

**Response:** This suggestion is crucial for us to improve our paper. In lines 380 to 384, we modified our statement so that we only base our argument on the results of empirical analysis, arguing that the public who use the Internet has lower political trust, and Hypothesis 2 is established.

**Comment 3 (Reviewer #1):** “The level of public political trust in respondents who never use the Internet is much higher than that in people who use the Internet several times a week and every day”. As the refence (baseline) category is ‘every day’ you can only say something about ‘every day’ vs. ‘several times a week’ and ‘every day’ vs. ‘never’. You cannot compare ‘never’ vs. ‘several times a week’ as neither of these categories is the reference category.

**Response:** We fully agree with this amendment. We only compare other categories with ‘every day’ to observe the significance and coefficients, and then determine the impact of Internet use frequency on political trust.

**Comment 4 (Reviewer #1):** “This may be attributed to the public’s heightened focus on economic development, driven by the growth of China’s socialist market economy”. There are no sources or data presented that support this claim. If making such a statement, it would be good to reference to sources.

**Response:** This comment helped us discover our omissions. We first added a reference to support our view in line 414. We also added a note from line 414 to line 415 about how the CGSS survey format might affect the public's response rate.

**Comment 5 (Reviewer #1):** “Specifically, exposure to news on the internet significantly promotes political participation, while recreational use of the internet negatively affects political participation. Thus, this paper further enriches the understanding of how internet use influences political apathy.” The paper does not measure content of internet usage and also does not measure the concept of political apathy, so these conclusions are not supported by the data.

**Response:** We support this amendment. We have made modifications from lines 436 to 438. After introducing the concept of political apathy, we directly point out that this study found that whether or not to use the Internet and the frequency of Internet use do not affect political apathy. This will help future researchers focus on the relationship between other areas such as Internet usage preferences and political apathy.

**Comment 6 (Reviewer #1):** “Third, this study also validates the perspective that even with the establishment of a well-developed gatekeeping system, prolonged exposure to positive information on the internet can still reduce political trust”. Again, the paper does not measure content of internet usage, so this conclusion cannot be derived from the data.

Response: After carefully considering the experts' suggestions, we found that our article results could not lead to such a conclusion. In order to ensure the scientificity and rigor of the research paper, we directly deleted this statement in line 545.

**Comment 7 (Reviewer #1):** “It is necessary to severely punish illegal acts that may affect public political participation, and to ensure the public’s successful performance of their own political rights.” This is quite a strong statement, how are ‘illegal acts’ defined and what is understood by ‘severely punish’?

**Response:** This comment does point out our problem, but it is difficult to clearly define its scope within the limited space. Therefore, we have directly deleted this statement in line 485 and directly proposed some feasible suggestions.

**Comment 8 (Reviewer #1):** “The terminology ‘political trust choice’ is a bit confusing. It would be good to rename this variable to something that makes it clear what is measured. For instance, ‘answer political trust’.”

**Response:** We fully agree with the expert’s opinion and we have changed ‘political trust choice’ to ‘answer political trust’.

**Comment 9 (Reviewer #1):** The authors could provide more information on the CGSS. For instance, how is the survey conducted (telephone, digital, by mail, in person etc.)? Is participation anonymous? This may impact why respondents do or do not answer the question about political trust.

**Response:** The author retrieved relevant news reports on CGSS2021 and determined the data obtained through home visits adopted by CGSS2021. We have also provided the source of the relevant news reports in the form of footnotes on page 6, which will help readers have a deeper understanding of this project.

**Comment 10 (Reviewer #1):** “Effect sizes can be presented to illustrate the impact of the significant results that are found.”

**Response:** This is an important observation. We report the regression coefficients, exp(B), and other values in the binary logistic analysis to make the results more visible. At the same time, we report indicators such as estimated values in the ordinal regression analysis.

**Comment 11 (Reviewer #1):** “The layout of the tables (especially 4 and 6) should be changed, as the tables are currently difficult to read. For instance, by presenting one single column of variables on the left, adding model x and model y as columns and presenting the coefficients and standard errors for each variable in each model. The significance levels can be presented as stars (* p<0.05 ** p<0.01 *** p<0.001).”

**Response:** This comment was crucial in improving our table layout. We adjusted Tables 4 and 6 accordingly, placing the variables on the left and Model x and Model y in the columns. Standard errors were placed within the regression coefficients or estimates, presented separately in parentheses. Significance was also indicated with “*”. This made the tables more visually appealing and intuitive, helping readers better understand the empirical results.

**Comment 12 (Reviewer #1):** “It looks like the dependent variables in Table 4 are continuous, while the same dependent variables in Table 6 are categorical. Why is that?”

**Response:** Dear experts! Table 4 presents the impact of internet use on "answer political trust." "Answer political trust" is a dichotomous variable, categorized as 0 or 1, and therefore represents the results of a binary logistic analysis. Table 6, on the other hand, presents the impact of internet use on the level of political trust, which is categorized as 1, 2, and 3. It also presents the results of an ordinal regression analysis.

**Comment 13 (Reviewer #1):** “It looks like the dependent variables in Table 4 are continuous, while the same dependent variables in Table 6 are categorical. Why is that?”

**Response:** Dear experts! Table 3 presents the prerequisite results for whether a binary logistic analysis can be conducted. If the relevant indicators in Table 3 are met, a binary logistic analysis can be conducted. We initially intended to combine the results of Tables 3 and 4, but considering that the combined results would be too large, we have to present the results in two tables. Therefore, Tables 3 and 4 present the results of Model 1 and Model 2. Table 5 reports the premises for conducting an ordinal regression analysis, and Table 6 reports the results of an ordinal regression analysis of the impact of internet use on political trust. Both Tables 5 and 6 present the results of Models 3 and 4.

**Comment 14 (Reviewer #1):** “The Chinese People’s Congress system is an effective form of political organization that is in line with China’s national conditions”. What does this mean?

**Response:** We have modified the sentence from lines 47 to 49, and we describe it as a political system arrangement that conforms to China's national conditions.

**Comment 15 (Reviewer #1):** “Clicks, likes, and shares lead to personalized recommendations that challenge the beliefs of users.” Is it challenge or affirm the beliefs of users?

**Response:** We would like to thank the expert for pointing out this error. After checking the references, we found that it does indeed affirm the user's idea. We have made changes in line 225.

**Comment 16 (Reviewer #1):** “typo, two periods/full stops.”

**Response:** We would like to thank the expert for pointing out our spelling error. We have deleted the redundant symbols in line 101 and checked the rest of the text.

Finally, we would like to thank the experts again for their efforts in improving this paper and enhancing its quality.
