# Supplementary material for: The impact of internet use in the digital era on public political trust in China—An empirical study based on CGSS 2021 data
Source: PLoS One. 2025 Dec 31;20(12):e0339999. doi: 10.1371/journal.pone.0339999 (PMC12755784; doi:10.1371/journal.pone.0339999)
Supplement: Attachment — Submitted filename: 20251025[Response to Reviewers].docx [file pone.0339999.s004.docx]

Rebuttal Letter for Manuscript ID: PONE-D-25-01950R1

Part 1: Cover Letter to the Editor

Revised Manuscript Submission (Manuscript ID: PONE-D-25-01950R1) - Response to Reviewers' Comments

Dear editor,

Thank you for giving us the opportunity to revise your manuscript entitled “The Impact of Internet Use in the Digital Era on Public Political Trust in China—An Empirical Study Based on CGSS 2021 Data” (Manuscript ID: PONE-D-25-01950R1]. We sincerely appreciate the time and effort invested by you and the reviewers in evaluating our work. We found the reviewers' comments to be constructive, insightful, and invaluable for improving the quality and impact of our paper.

We have carefully considered all points raised by the reviewers and have made extensive revisions to the manuscript accordingly. A detailed, point-by-point response to each specific comment from each reviewer is provided in the attached document. The main categories of revisions we have undertaken include:

Based on the actual situation of this survey and considering that it is inappropriate to use the term "political apathy" in the article, we deleted the expression of political indifference in the article. Instead, based on the existing literature that Internet use affects interpersonal trust and trust affects the willingness to share personal information, we re-organized the "research hypothesis" part and the "discussion and conclusion" part of the article.

Comprehensive Reference Updates: References have been checked, updated where necessary, and formatted according to journal guidelines. Our commentary on cited works is now more substantial and critical.

All changes in the revised manuscript were tracked using the Track Changes feature in Microsoft Word. We firmly believe that these revisions significantly improved the manuscript's quality in terms of language, clarity, scientific depth, logical structure, presentation of figures and tables, and accuracy of references, effectively addressing the core concerns raised by the reviewers.

We respectfully request that you reconsider our revised manuscript for publication in PLOS ONE.

We are fully committed to further refining this work and are more than willing to undertake any additional revisions deemed necessary to meet the high standards of this esteemed journal.

Thank you again for your time and consideration. We look forward to your further guidance.

Sincerely,

Lu Fang

On behalf of all co-authors

University of Electronic Science and Technology of China

Part 2: Detailed Point-by-Point Response to Reviewers' Comments

Manuscript ID: PONE-D-25-01950R1

Title: The Impact of Internet Use in the Digital Era on Public Political Trust in China—An Empirical Study Based on CGSS 2021 Data

Response to Reviewer #1 Comments

We thank Reviewer #1 for their careful reading and constructive suggestions, which have greatly helped us improve the manuscript. Below are our point-by-point responses. All modifications are made using revision mode.

**Comment 1 (Reviewer #1):** “Thank you for the opportunity to review the revised version of “The impact of internet use in the digital era on public political trust in China”. The authors have addressed several of the concerns that were raised in the first round of review. I commend the authors for the work that they did. However, I believe there still are some issues that make that at this stage the paper is not ready for publication.

An important issue is that there is a disconnect between the argument on political apathy and the results and conclusions that are presented. The concept of political apathy plays a central role in the conclusions presented by the authors, but from the theoretical and methods section it is not clear how political apathy is conceptualized. The authors argue that political trust manifests at a deeper level as political apathy, and that therefore not answering a survey question about political trust is an indicator of political apathy. This argument requires clarification and elaboration to convince the reader why this is an indicator of political apathy.

The authors have made adjustments to clarify hypotheses one and three, but the link between internet usage and not answering a question about political trust remains unclear. The concept of political participation is introduced, and it is stated that political participation has changed due to internet usage. However, the authors then argue that people may be less likely to answer questions about political trust due to the in-person interviews (as they would be more likely to want to protect their privacy due to internet usage). But the question that emerges is: do people not answer the question due to their internet usage or due to the way in which the data was collected (in-person interviews)?

It is very informative that the authors have added information about the sample collection methods of the CGSS2021 survey. However, the fact that the survey was conducted by means of in-person interviews at home, might be a quite impactful variable (as the authors mention that questions about political trust can be regarded as sensitive). Based on the argumentation the link between internet usage and not answering questions about political trust remains vague. I believe the questions should be: are people more likely to protect their privacy due to internet usage? If they do, it can be expected that they are more likely not to be willing to answer questions about political trust during in-person interviews.

In sum, the authors have to make explicit why not answering questions about political trust is an indicator of political apathy. In addition, the authors have to provide a convincing case on how internet usage affects not answering political trust questions and how they measure this impact.”

**Response:** We are very grateful for pointing out the problems with our article and we fully agree with it. Based on your comments, combined with a review of relevant literature and the CGSS survey methodology, we have abandoned our previous interpretation of not answering the political trust question as political apathy and answering it as political participation. This is inappropriate given the current data and the definitions of political apathy and political participation.

Your suggestion to modify the article from a privacy perspective provides us with a good idea. First, in the research hypothesis section, we combined Hypotheses 1 and 3, which originally addressed answering the political trust question, and renumbered Hypothesis 3 as Hypothesis 2. We also added the following before Hypotheses 1 and 2:

“According to relevant CGSS reports^[1]^, the 2021 CGSS employed in-home interviews for data collection. This raises the issue of the respondents' trust in the interviewers^[2]^. Interpersonal trust encompasses multiple dimensions, ranging from trust in relatives, friends, and acquaintances to trust in strangers[32]. Interpersonal trust entails positive expectations regarding the behavior of others, thereby motivating individuals to engage in interactions through sharing, mutual assistance, and cooperation[33], while also enhancing their willingness to disclose personal information[34]. Trust is essentially the willingness to take the risk of information disclosure[35]. Political trust refers to the public's subjective evaluation and attitude toward political institutions and politicians[36]. An article on the official website of the Cyberspace Administration of China classifies political opinions and other personal information as sensitive[37]. Similarly, the European Union's *General Data Protection Regulation* (GDPR) specifies that individuals' political opinions constitute special categories of personal data[38]. Thus, from this vantage point, individuals' responses to questions regarding political trust fall within the scope of personal privacy.

However, Chinese scholars have utilized data from the CGSS 2013 and CGSS 2015 surveys to demonstrate that Internet use exerts a significant negative effect on public trust in strangers[39]. Internet use diminishes respondents' trust in interviewers, thereby reducing the propensity for sharing, mutual assistance, and cooperation between them. Trust fundamentally entails the willingness to assume the risks associated with information disclosure[35]; consequently, owing to the absence of trust between them, respondents are reluctant to respond to questions pertaining to political trust. Drawing on the foregoing analysis, we advance the following two hypotheses.”

Now, I'd like to share with you my modification ideas. After reviewing relevant laws and regulations, we found that personal political views are indeed sensitive personal information. Therefore, answering questions about political trust involves the disclosure of personal privacy. A review of the literature on personal privacy disclosure reveals that trust and perceived risk are important factors. Existing research has shown that internet use affects interpersonal trust, with internet use significantly negatively impacting trust in strangers. Furthermore, trust is essentially an individual's willingness to assume the risk of disclosing information. News reports on the CGSS survey suggest a lack of trust between respondents and interviewers, leading to a potential reluctance to assume the risk of disclosing information. Based on this, we propose Hypotheses 1 and 2.

The “implications for research” section in the “discussion and conclusions” of the article, we also deleted the previous content on political apathy and political participation. Instead, we discussed Internet use, interpersonal trust, and private information disclosure.

In the “Implications for practice” section, we rewrote the second paragraph, which discusses research methods and approaches to ensure that the public is more willing to answer questions about political trust.

Finally, we thank you again for your efforts in improving this article and enhancing its quality.

1. For related news reports, see "Summer Semester 100 Village Survey: 2021 Chinese General Social Survey (CGSS)" at: <https://spm.ncu.edu.cn/tsgz/bcdc/1c11c5be93544dfc9cc3935d8ab9d635.htm;>

   "The China Survey and Data Center 2021 Report Card is released!" The website is: https://mp.weixin.qq.com/s?__biz=MzU3NTAwNjk4MA==&mid=2247493929&idx=1&sn=58a9b87ec3dee244e37a0f468e7b37a5&chksm=fd2b1942ca5c9054b7e853ad6226df4b3325b72ee8e763c0581f6960dd26d2a2fdb39248d4e7&scene=27.
2. For related news reports, see "Summer Semester 100 Village Survey: 2021 Chinese General Social Survey (CGSS)" at: https://spm.ncu.edu.cn/tsgz/bcdc/1c11c5be93544dfc9cc3935d8ab9d635.htm.
