# Supplementary material for: The impact of internet use in the digital era on public political trust in China—An empirical study based on CGSS 2021 data
Source: PLoS One. 2025 Dec 31;20(12):e0339999. doi: 10.1371/journal.pone.0339999 (PMC12755784; doi:10.1371/journal.pone.0339999)
Supplement: Attachment — Submitted filename: 20251125[Response to Reviewers].docx [file pone.0339999.s005.docx]

Rebuttal Letter for Manuscript ID: PONE-D-25-01950R2

Part 1: Cover Letter to the Editor

Revised Manuscript Submission (Manuscript ID: PONE-D-25-01950R2) - Response to Reviewers' Comments

Dear editor,

Thank you for giving us the opportunity to revise your manuscript entitled “The Impact of Internet Use in the Digital Era on Public Political Trust in China—An Empirical Study Based on CGSS 2021 Data” (Manuscript ID: PONE-D-25-01950R2]. We sincerely appreciate the time and effort invested by you and the reviewers in evaluating our work. Over the course of more than 10 months, you and the reviewers provided three rounds of revision opportunities and feedback, which helped us improve the quality of this paper. We found the reviewers' comments to be constructive, insightful, and invaluable for improving the quality and impact of our paper.

We have carefully considered all points raised by the reviewers and have made extensive revisions to the manuscript accordingly. A detailed, point-by-point response to each specific comment from each reviewer is provided in the attached document. The main categories of revisions we have undertaken include:

Since the previously uploaded data was in SAV format, it requires SPSS software to open. To facilitate editorial review and reader downloads, we have uploaded an additional dataset in XLSX format based on the SAV dataset. Both datasets contain identical data.

The S2 data we provide does not involve any disclosure of personal privacy. This data originates from China's official public database, with data collection strictly adhering to Chinese law. The publicly available data has undergone anonymization processing. Authors using this database to write papers do not require additional approval from ethics committees.

When uploading manuscripts to the system, we modified the manuscript titles within the system to align with the document titles.

We have removed all references to funding information and financial support from the document.

We made targeted and comprehensive revisions based on several questions raised by the reviewers.

We have incorporated the footnotes into the reference list and modified the reference format according to the PLOS ONE journal style requirements.

At the same time, we reviewed the entire text again and adjusted some sentences and wording to ensure there were no grammatical or semantic errors.

All changes in the revised manuscript were tracked using the Track Changes feature in Microsoft Word. We firmly believe that these revisions significantly improved the manuscript's quality in terms of language, clarity, scientific depth, logical structure, presentation of figures and tables, and accuracy of references, effectively addressing the core concerns raised by the reviewers.

We respectfully request that you reconsider our revised manuscript for publication in PLOS ONE.

We are fully committed to further refining this work and are more than willing to undertake any additional revisions deemed necessary to meet the high standards of this esteemed journal.

Thank you again for your time and consideration. We look forward to your further guidance.

Kind regards,

Lu Fang

On behalf of all co-authors

University of Electronic Science and Technology of China

Part 2: Detailed Point-by-Point Response to Reviewers' Comments

Manuscript ID: PONE-D-25-01950R2

Title: The Impact of Internet Use in the Digital Era on Public Political Trust in China—An Empirical Study Based on CGSS 2021 Data

Response to Reviewer #1 Comments

We thank Reviewer #1 for their careful reading and constructive suggestions, which have greatly helped us improve the manuscript. Below are our point-by-point responses. All modifications are made using revision mode.

**Comment 1 (Reviewer #1):** Throughout the paper the authors deploy the terms "choice of political trust" and "answer political trust". These terms are confusing as it is not clear what these concepts entail. The authors should either replace these terms (for instance: responsiveness to political trust questions) or define "choice of political trust"/"answer political trust" from the start.

**Response 1:** We highly appreciate the reviewer's comment, as it is of great help in improving our paper and in assisting readers. We adopted the reviewers' suggestions to replace the terms "choice of political trust" and "answer political trust" with "responsiveness to political trust questions". We added "This necessitates that this article pay attention to questions about public responsiveness to political trust. Therefore, this paper examines the impact of internet use on public political trust, encompassing both its influence on responsiveness to political trust questions and its effect on the overall level of public political trust." after "At the same time, an interesting phenomenon was also discovered, namely that among the total sample of 8,148 respondents, only 2,613 responded to relevant questions, while nearly 2/3 of all respondents did not respond this question." In order to help readers better understand responsiveness to political trust.

**Comment 2 (Reviewer #1):** Line 111-115: this section discusses the impact of factors on political trust. One of these factors is described as ‘income’ ("… income reduces trust in mature democracies"). Shouldn’t this be income level? And is it then that lower or higher income levels reduce political trust?

**Response 2:** We appreciate your meticulous pointing out of this issue. We reread the paper containing this viewpoint, and indeed, income here refers to income level, and in mature democracies, high income levels can actually lead to low political trust. At the same time, we have made corresponding modifications to the original text, changing it to "on the contrary, post-materialism, political radicalism, corruption indulgence, and income level impede political trust[21]. In other words, in mature democracies, the higher an individual's income level, the lower their level of political trust. At the same time, income level has different effects in different countries. While income level reduces trust in mature democracies, it increases trust in countries of Latin America and Eastern Europe, but has no effect on the countries of the former Soviet Union[21]."

**Comment 3 (Reviewer #1):** Line 143-144: "In the new information environment created by the Internet, political trust has been strengthened…" Has should be can

**Response 3:** Thank you for your suggestion. We have revised the original text from "In the new information environment created by the Internet, political trust has been strengthened" to "In the new information environment created by the Internet, political trust can been strengthened’

**Comment 4 (Reviewer #1):** Line 346: "average education level is 9.31, and the average is 9,…" Should be "and the median is 9"

**Response 4:** Thank you for pointing out this error. We located the original text and changed the average to the median.

**Comment 5 (Reviewer #1):** Line 380-384: "level of political trust in people/respondents" should be "level of political trust among people/respondents"

**Response 5:** Thank you so much for pointing out this error. We have corrected that part of the original text.

**Comment 6 (Reviewer #1):** In the discussion section some additional findings are discussed. Additional finding 2 and 3 do not seem to be derived from the data analyses, but rather seem to be conclusions based on literature research. If this is not the case and these numbers are actually derived from the data analyses, the authors should discuss these findings in the data section.

**Response 6:** Thank you for your suggestion. Indeed, we should not draw upon viewpoints from other literature here. We have made the following adjustments.

First, we removed the four additional findings from the original proposal and retained only the findings obtained from the article's data analysis. We changed the value after "...while those who use the internet a few times a week demonstrate the lowest level" to "At the same time, the study also found that religious belief significantly influence public's responsiveness to political trust questions; those with religious beliefs are even less likely to respond to such questions. Furthermore, there are significant gender differences in the level of political trust among the public, with men exhibiting significantly higher levels of political trust than women".

Second, in the section "The impact of Internet use on "responsiveness to political trust questions" of the public". We added "Among the control variables, we found that the public's religious belief had a significant negative impact on responsiveness to political trust questions, while age, political status, education level, and socioeconomic status had no significant impact" after "it is assumed that hypothesis 2 is not verified".

Third, in the section "The impact of public Internet use on the level of political trust". We added "Among the control variables, there was a significant gender difference in the public's level of political trust in the National People's Congress, with men showing higher levels of trust than women. Other control variables did not have a significant impact on the level of public political trust" after "This result shows that the impact of the frequency of public Internet use on political trust is not linear".

Finally, we read the entire text again and, without changing the existing structure and content, we adjusted some sentences to ensure there were no grammatical or spelling errors.

We thank you again for your efforts in improving this article and enhancing its quality.

Kind regards.
